# Racial Disparity in Prostate Cancer-Specific Mortality for High-Risk Prostate Cancer: A Population-Based Study

**DOI:** 10.7759/cureus.961

**Published:** 2017-01-06

**Authors:** Chenyang Wang, Mitchell Kamrava, Chris King, Michael L. Steinberg

**Affiliations:** 1 Department of Radiation Oncology, UCLA Medical Center

**Keywords:** prostate cancer, radiotherapy, brachytherapy, dose-escalation, race

## Abstract

**Introduction:**

Race may be a significant factor that influences prostate cancer (PCa) survival, with the Asian (AsA) race being associated with better outcomes compared to African American (AA) and Non-Hispanic Whites (NHW). This study evaluates race-dependent variation in PCa-specific mortality (PCSM) associated with radiation dose-escalation exemplified by external beam radiotherapy (EBRT) with a brachytherapy (BT) boost in Gleason score 8-10 PCa.

**Methods:**

28,956 men diagnosed with clinically localized PCa and Gleason score 8-10 from 2004–2013 who received EBRT, EBRT with a BT boost, or radical prostatectomy (RP) were identified using the Surveillance, Epidemiology, and End Results (SEER) database. PCSM adjusted for age, diagnosis year, T-stage, Gleason scores, and treatment modalities was compared between races using a competing risk model that accounted for other-cause mortality (OCM).

**Results:**

Compared to AsA, AA and NHW are associated with significantly increased PCSM with adjusted hazard ratios (AHR) of 2.295 and 1.989, respectively (p < 0.001 for both). In a subgroup analysis stratified by race, dose-escalation exemplified by EBRT with a BT boost in both AA and AsA failed to demonstrate a significant difference in PCSM compared to EBRT alone (p = 0.530 and 0.990, respectively), while a significant PCSM decrease was seen in NHW (p = 0.006).

**Conclusions:**

Dose-escalation exemplified by EBRT with a BT boost had no significant effect on PCSM of AsA and AA, while it did decrease PCSM amongst NHW. Further evaluation of race as a factor impacting PCSM in the era of dose-escalation is needed in the prospective setting.

## Introduction

There is increasing awareness that race plays an important role in prostate cancer (PCa) survival. A Surveillance, Epidemiology, and End Results (SEER)-Medicare linked analysis of men with advanced stage PCa showed no statistically significant difference in disease-specific survival (DSS) between Non-Hispanic Whites (NHW) and African Americans (AA), while Asian Americans (AsA) demonstrated superior DSS compared to NHW [1]. Another SEER analysis comparing AsA and NHW with localized (TxN0M0), regional (TxN1M0) and metastatic (TxNxM1) PCa concluded that AsA demonstrate improved DSS despite being more likely to present with advanced PCa [2]. The underlying hypothesis for this race-dependent variation in PCa survival is unclear and likely multifactorial in nature.

Along with the recognition of race as a possible prognostic factor in PCa, there is also growing evidence that dose-escalated radiotherapy (RT) is associated with improved disease progression and possibly survival [3-5]. This is supported by a body of level two evidence demonstrating superior outcomes with a combination of external beam radiotherapy (EBRT), a brachytherapy (BT) boost, and androgen deprivation therapy (ADT). It is also supported by level one evidence from the Androgen Suppression Combined With Elective Nodal and Dose Escalated Radiation Therapy (ASCENDE-RT) trial in unfavorable intermediate and high-risk patients showing approximately a 20% improvement in prostate-specific antigen (PSA) biochemical control at nine years in patients treated with EBRT and a BT boost (EBRT+BT) compared with EBRT alone [6]. We await long term results from ASCENDE-RT to see if the improvement in PSA control will translate into reduced distant metastasis and possible improvement in overall survival. While these results are promising, the improvement in disease progression with dose-escalation in the ASCENDE-RT trial came at the cost of increased toxicity. The cumulative five-year genitourinary (GU) grade 3 toxicities was 19% versus five percent, and the prevalence of late grade 3 or higher toxicity was eight percent vs. two percent for the EBRT+BT arm versus the EBRT alone arm. So while dose-escalation can improve PCa outcomes, it is important to determine whether all patients benefit from this treatment as it can come at the cost of increased toxicity.

In this study, our primary hypothesis is that race is a factor that influences the survival benefit associated with dose-escalated RT. Given the lack of prospective or retrospective studies adequately powered to investigate the impact of race on prostate cancer-specific mortality (PCSM), we used the SEER database to investigate the impact of dose-escalation exemplified by EBRT+BT on PCSM stratified by race.

## Materials and methods

### Patient population

For this study, we selected patients with clinically localized PCa staged as any T stage N0M0 with Gleason score 8-10 diagnosed between January 1st, 2004 and December 31st, 2013. This study focused on high-risk PCa patients with Gleason score 8-10 due to the increased likelihood of this cohort to experience PCSM during the study period, which will help to reveal any significant relationships between study covariates and PCSM. Patients who matched these selection criteria were extracted from the SEER database for subsequent analysis. Patients who underwent surgeries other than RP, had non-adenocarcinoma histology, were not in active follow-up, had more than one primary malignancy and/or had age younger than 18 years at diagnosis were excluded from further analysis.

Definitive treatments were defined as RP, EBRT, and EBRT+BT. We also separately selected patients who received no definitive treatment (defined as no surgery or RT as the first course of treatment) to evaluate the approximate natural progression of high-risk PCa. This study’s start date was chosen because of the sharp rise in intensity modulated radiotherapy (IMRT) utilization for PCa starting in 2002 [7], in order to homogenize the EBRT technique and dose. All Gleason scores used in this analysis were determined from biopsy in order to reduce the potential bias of Gleason score upgrading based on final surgical pathology, and T stage was determined clinically. This study was reviewed and approved by the institutional review board at the University of California, Los Angeles.

The SEER database currently does not have quantitative PSA data due to a recently discovered error, which resulted in a misplacement of a decimal point affecting approximately three to four percent of the patients [8].

### Statistical analysis

The variables evaluated include age, year of diagnosis, race, Gleason score, T stage, and definitive treatment modalities. Analysis of variance (ANOVA) was used to characterize difference in age between cohorts defined by race, whereas chi-square test was used to characterize differences in year of diagnosis, Gleason score, T stage and definitive treatment modality. The median follow-up time was calculated via the reverse Kaplan-Meier method described by Schemper and Smith, et al. [9], in which 'alive at last follow-up' was designated the event of interest while 'death from any cause' was censored.

Because of the overall slow progression of PCa, significantly more patients in the cohort died of causes other than PCa compared to PCa itself (1,980 vs. 1,326). This other-cause mortality (OCM) is often secondary to other medical comorbidities, which has varying distributions associated with race or definitive treatment modalities. In survival analysis, OCM prevents PCSM from being observed, and therefore OCM serves as a competing risk for PCSM. In a standard Cox proportional hazards survival model, OCM is censored along with patients who were alive at last follow-up, which implicitly ignores OCM. In order to appropriately account for OCM as a competing risk for PCSM during our survival analysis, multivariate regression analysis based on the proportional hazards model described by Fine and Gray was applied to the data [10], while adjusting for covariates including age, year of diagnosis, Gleason score, T stage, and definitive treatment modality. The patients who were alive at last follow-up were censored. A subgroup multivariate regression analysis stratified by race was also carried out. Competing risk (PCSM vs. OCM) estimates of the cumulative incidence function were calculated and plotted after stratification according to race for patients who underwent definitive treatments and also separately for patients who underwent no definitive treatment. All p-values were two-sided with a statistical significance threshold of 0.05. All statistical analyses were carried out using R version 3.3.1 (R Foundation for Statistical Computing, Vienna, Austria).

## Results

We identified 28,956 patients diagnosed between 2004 and 2013 with clinically localized (any T stage N0M0) PCa with Gleason score 8-10 matching our selection criteria. There were statistically significant differences in age, year of diagnosis, Gleason score, T stage and definitive treatment modalities between the races as shown in Table 1. Of note, AsA tend to be older at the time of diagnosis with a median age of 69 years, followed by NHW at 68 years and AA at 64 years. AA tend to present with lower T stage and Gleason score (Gleason 8) compared to AsA and NHW. They were also more likely to undergo EBRT instead of RP for definitive treatment. Median follow-up calculated via the reverse Kaplan-Meier method was 54 months for all three races.

**Table 1:** Baseline patient characteristics.

	AsA	AA	NHW	p
N	2253	4712	21991	
Age (median [IQR])	69.00 [63.00, 74.00]	64.00 [59.00, 70.00]	68.00 [62.00, 74.00]	<0.001
Year of Diagnosis (%)				0.002
2004	161 (7.1)	336 (7.1)	1744 (7.9)	
2005	180 (8.0)	349 (7.4)	1692 (7.7)	
2006	206 (9.1)	400 (8.5)	2139 (9.7)	
2007	228 (10.1)	451 (9.6)	2322 (10.6)	
2008	230 (10.2)	509 (10.8)	2233 (10.2)	
2009	236 (10.5)	431 (9.1)	1931 (8.8)	
2010	228 (10.1)	551 (11.7)	2580 (11.7)	
2011	304 (13.5)	585 (12.4)	2574 (11.7)	
2012	244 (10.8)	550 (11.7)	2358 (10.7)	
2013	236 (10.5)	550 (11.7)	2418 (11.0)	
Gleason Score (%)				<0.001
8	1366 (60.6)	3136 (66.6)	13354 (60.7)	
9	826 (36.7)	1461 (31.0)	8029 (36.5)	
10	61 (2.7)	115 (2.4)	608 (2.8)	
T_stage (%)				<0.001
T1	1143 (50.7)	2817 (59.8)	10551 (48.0)	
T2	922 (40.9)	1583 (33.6)	9634 (43.8)	
T3	176 (7.8)	278 (5.9)	1671 (7.6)	
T4	12 (0.5)	34 (0.7)	135 (0.6)	
Treatment (%)				<0.001
EBRT+BT	269 (11.9)	545 (11.6)	2214 (10.1)	
EBRT	1034 (45.9)	2407 (51.1)	9920 (45.1)	
RP	950 (42.2)	1760 (37.4)	9857 (44.8)	

Multivariate regression analysis for PCSM was carried out to explore the impact of age, year of diagnosis, race, Gleason score, T stage and definitive treatment modality on PCSM. The results in Table 2 show that higher Gleason score and T stage are associated with increased PCSM. Using EBRT+BT as the reference group, patients treated with RP demonstrated significantly decreased PCSM with an adjusted hazard ratio (AHR) of 0.766 (p = 0.007) while those treated with EBRT alone demonstrated significantly increased PCSM with an AHR of 1.268 (p = 0.010). Finally, AA and NHW races were associated with significantly increased PCSM compared to AsA, with AHRs of 2.295 (p < 0.001) and 1.989 (p < 0.001), respectively.

**Table 2:** Multivariate regression analysis for PCSM using the proportional hazards model described by Fine and Gray, accounting for OCM as a competing risk for PCSM.

	AHR [95% CI]	p
Gleason Score:		
8	1.000	
9	2.146 [1.913-2.406]	<0.001
10	4.402 [3.539-5.474]	<0.001
Race:		
AsA	1.000	
AA	2.295 [1.708-3.083]	<0.001
NHW	1.989 [1.519-2.605]	<0.001
Treatment:		
EBRT+BT	1.000	
RP	0.766 [0.630-0.931]	0.007
EBRT	1.268 [1.059-1.517]	0.010
T stage:		
T1	1.000	
T2	1.234 [1.095-1.392]	0.001
T3	1.998 [1.682-2.373]	<0.001
T4	3.158 [2.147-4.645]	<0.001
Age at diagnosis:	1.005 [0.998-1.013]	0.150
Year of diagnosis:	0.913 [0.888-0.939]	<0.001

Subgroup multivariate regression analysis for PCSM was subsequently carried out for each race, with the results shown in Table 3. AsA demonstrated non-significant differences in PCSM between EBRT+BT and RP (p = 0.800). EBRT also resulted in equivalent PCSM compared to EBRT+BT (p = 0.530). Meanwhile, NHW demonstrated a trend for decreased PCSM with RP compared to EBRT+BT with an AHR of 0.810 (p = 0.065), and EBRT is associated with significantly increased PCSM compared to EBRT+BT with an AHR of 1.338 (p = 0.006). Amongst AA, RP demonstrated significantly decreased PCSM compared to EBRT+BT with an AHR of 0.596 (p = 0.023), while EBRT resulted in equivalent PCSM as EBRT+BT with an AHR of 0.997 (p = 0.990).

**Table 3:** Subgroup multivariate regression analysis for PCSM for each race, using the proportional hazards model described by Fine and Gray, accounting for OCM as a competing risk for PCSM.

	AsA		AA		NHW	
	AHR [95% CI]	p	AHR [95% CI]	p	AHR [95% CI]	p
Gleason Score:						
8	1.000		1.000		1.000	
9	2.005 [1.176-3.420]	0.011	2.821 [2.138-3.723]	<0.001	2.024 [1.779-2.302]	<0.001
10	2.287 [0.637-8.220]	0.200	5.051 [3.017-8.455]	<0.001	4.448 [3.479-5.687]	<0.001
Treatment:						
EBRT+BT	1.000		1.000		1.000	
RP	0.894 [0.372-2.150]	0.800	0.596 [0.382-0.931]	0.023	0.810 [0.648-1.013]	0.065
EBRT	1.307 [0.563-3.030]	0.530	0.997 [0.670-1.486]	0.990	1.338 [1.087-1.646]	0.006
T stage:						
T1	1.000		1.000		1.000	
T2	1.631 [0.917-2.900]	0.096	1.193 [0.892-1.596]	0.240	1.225 [1.070-1.403]	0.003
T3	1.400 [0.505-3.880]	0.520	2.426 [1.637-3.594]	<0.001	1.956 [1.610-2.377]	<0.001
T4	6.214 [1.731-22.31]	0.005	2.688 [1.237-5.842]	0.013	3.149 [1.961-5.056]	<0.001
Age at diagnosis:	1.002 [0.968-1.040]	0.930	1.000 [0.982-1.018]	1.000	1.007 [0.998-1.015]	0.120
Year of diagnosis:	0.892 [0.758-1.050]	0.170	0.952 [0.888-1.020]	0.160	0.906 [0.878-0.934]	<0.001

**Figure 1:**
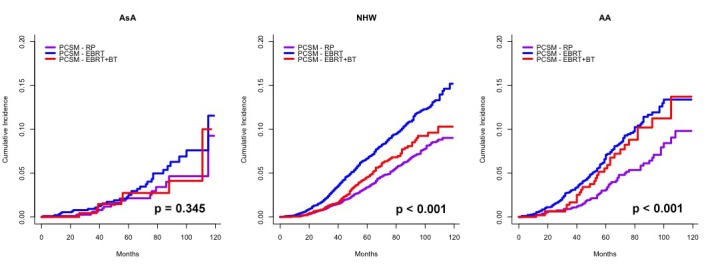
Subgroup cumulative incidence of PCSM based on the proportional hazards model described by Fine and Gray, accounting for OCM as a competing risk for PCSM for each race and stratified by definitive treatment modality.

The cumulative incidence plots of PCSM and OCM stratified by race in Figure 1 support the subgroup multivariate regression analysis results shown in Table 3, showing that PCSM does not differ significantly between definitive treatment modalities amongst AsA men (p = 0.345). NHW demonstrate lower PCSM with EBRT+BT compared to EBRT; however, the cumulative incidence of PCSM for EBRT+BT is still elevated compared to RP (p < 0.001). Lastly, AA men demonstrated equivalent PCSM with EBRT+BT compared to EBRT, and both are significantly elevated compared to RP (p < 0.001).

**Figure 2:**
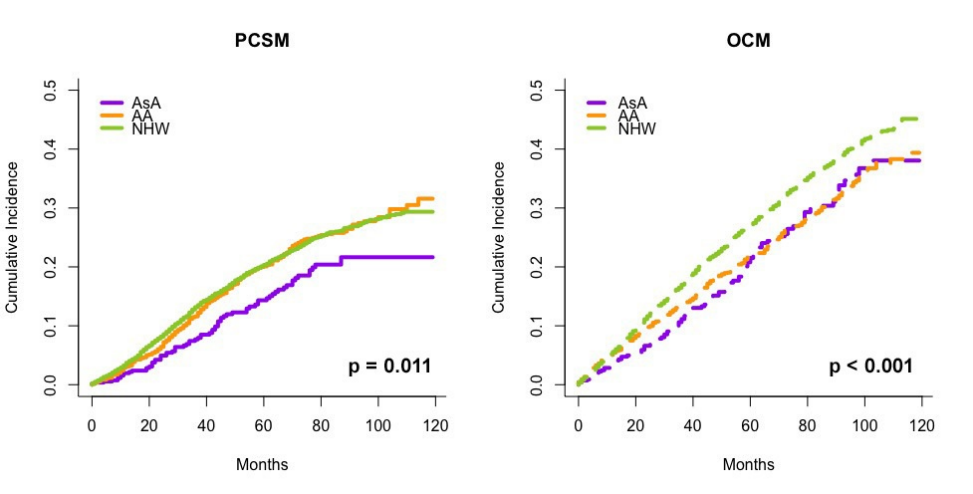
Cumulative incidence of PCSM (left) and OCM (right) for patients who received no definitive treatment stratified by race, calculated using the proportional hazards model described by Fine and Gray, accounting for OCM as a competing risk for PCSM.

We also separately evaluated PCSM of AsA, AA, and NHW men with clinical localized Gleason score 8-10 PCa (n = 572, 1,968 and 6,171, respectively) who did not undergo definitive treatment, in the presence of OCM as a competing risk. The resulting cumulative incidence plots of PCSM and OCM in Figure 2 show statistically significant lower PCSM for AsA men compared to AA and NHW men (p = 0.002).

## Discussion

Level one evidence from the ASCENDE-RT trial shows improved PSA control for patients treated with EBRT with a low-dose rate (LDR) BT boost compared to EBRT alone, with a nine-year PSA control of 83% vs. 63%, respectively [6]. We hypothesize that race may be a factor that influences PCa outcomes in patients who receive dose-escalated RT. In our study, we evaluated the impact of dose-escalation on PCSM for different races.

The subgroup multivariate regression analysis stratified by race revealed significant race-dependent variations in PCSM benefit in response to dose-escalation exemplified by EBRT+BT. In both AA and AsA, EBRT+BT failed to demonstrate a significant difference in PCSM compared to EBRT alone (p = 0.530 and 0.990, respectively), while EBRT for NHW was associated with a significantly increased PCSM compared to EBRT+BT with an AHR of 1.338 (p = 0.006). This observation of only NHW demonstrating a PCSM benefit with EBRT+BT is supported by the fact that more recent year of diagnosis in NHW is associated with a significantly decreased PCSM with an AHR of 0.906/year (p < 0.001), which we hypothesize reflects the increasing prevalence of dose-escalated EBRT. In comparison, more recent year of diagnosis for AsA and AA men non-significant (p = 0.170 and 0.160, respectively). Given dose-escalation via a BT boost is not associated with a significant PCSM benefit in AsA and AA in our study, it is hypothesis-generating for the need to re-evaluate whether high-risk PCa patients of all races should be similar candidates for dose-escalation via a BT boost. It is also not clear at this time what the mechanism is responsible for why some men would benefit in PCSM from dose-escalation via a BT boost whereas others would not. There is some data to suggest that there is biological basis to explain the tendency for more aggressive disease in AA, for example, but our current overall knowledge regarding this topic is limited [11-12]. A previous study has concluded that AA men are less likely to receive definitive treatment [13], and another study has concluded that AA men are less likely to receive RP if they were to undergo definitive treatment for PCa [14]. Similar patterns have been observed in our study, and they likely contributed and will continue to contribute to racial disparity in PCa survival negatively affecting AA men. The underlying cause for this race-dependent variation in PCSM benefit associated with dose-escalation is unclear, and it may be the result of a combination of socioeconomic [15], hormonal [16-17], dietary [18-19] and genetic factors [20].

Meanwhile, the ASCENDE-RT trial also demonstrated a higher incidence of grade 3 GU toxicities in patients who underwent a LDR BT boost [21]. While some of this increased toxicity may be related to the BT technique used, it nevertheless raises the question whether all patients benefit the same from dose-escalation, and if not whether some can avoid the potential increased toxicity associated with it.

Decreased PCSM in AsA compared to NHW and AA has been reported previously [1-2, 22]; however, no studies to the best of our knowledge have investigated the race-related differences in PCSM benefit associated with dose-escalation for localized PCa. In addition to the decreased PCSM of AsA compared to AA and NHW who underwent definitive treatments, we also observed decreased PCSM for AsA who did not undergo definitive treatment (log rank p = 0.011). This suggests that AsA may have a more favorable baseline PCa biology compared to other races.

This study has several limitations. First and foremost, accurate PSA information was not available in the SEER database prior to 2010 and neither was RT dose, EBRT modality (3D conformal RT vs. IMRT vs. stereotactic body RT) and BT modality (LDR vs. high dose rate). Another important limitation of the SEER database for PCa is the lack of ADT information. Several randomized trials have already demonstrated improved DSS with long-term ADT use in high-risk PCa [23-26]. Variations in ADT use such as omission or short versus long-term use could potentially affect PCSM; however, we do not expect significant systemic variation in ADT use between the treatment cohorts in our study. The SEER database also only records therapies delivered as the first course of treatment, and therefore it lacks information on recurrence and subsequent salvage treatment. Some investigators have suggested the standardization of a “multimodal” approach for high-risk PCa patients undergoing RP, which would include postoperative RT either in the adjuvant or salvage setting [27]. Notably, 1,967 of the 12,567 RP patients (15.7%) in this study cohort underwent RT as part of the “first course of treatment” following RP, likely representing a sizable proportion of patients receiving adjuvant RT. Although we cannot ascertain what proportion of RP patients in this study received RT in the salvage setting, it is likely that the majority of high-risk PCa patients who underwent RP failed to receive postoperative RT, consistent with health services studies in the United States showing that only <20% of patients with adverse pathologic features ultimately received postoperative RT [28]. As in the case of RT patients who received suboptimal radiation dose, RP patients who do not receive appropriate multimodal management potentially introduce bias into survival analyses. However, while dose-escalation and ADT use are considered standard of care especially for high-risk PCa, postoperative RT is still largely deferred at provider discretion [29]. Meanwhile, local salvage therapies after definitive RT are also not recorded and are known to be pursued even more infrequently than after RP [30]. Therefore, the deficiencies in SEER data collection does not impact any one particular definitive treatment modality, instead they are biased against all definitive treatment modalities.

## Conclusions

Our study confirmed that AsA race is associated with decreased PCSM for clinically localized high-risk PCa, after adjusting for age, Gleason score, T stage and definitive treatment modality. More importantly, our results showed race-dependent variations in PCSM benefit associated with dose-escalation exemplified by EBRT+BT, with AsA and AA failing to demonstrate significant decrease in PCSM with dose-escalation, while NHW men did. This finding of a race-dependent PCSM benefit due to dose-escalation is hypothesis-generating. Future prospective studies will need to place greater consideration on trial design to ensure that they are adequately powered to elucidate race’s impact on PCa outcome and to possibly explore individualized treatment options with the hope to close existing racial disparities in PCa survival.
